# The potential of fecal microbiota and amino acids to detect and monitor patients with adenoma

**DOI:** 10.1080/19490976.2022.2038863

**Published:** 2022-02-21

**Authors:** Sofie Bosch, Animesh Acharjee, Mohammed N Quraishi, Patricia Rojas, Abdellatif Bakkali, Erwin EW Jansen, Marina Brizzio Brentar, Johan Kuijvenhoven, Pieter Stokkers, Eduard Struys, Andrew D Beggs, Georgios V Gkoutos, Tim GJ de Meij, Nanne KH de Boer

**Affiliations:** a Amsterdam Umc, Vu University Medical Center, Department of Gastroenterology and Hepatology, Ag&m Research Institute, Amsterdam, The Netherlands; b College of Medical and Dental Sciences, Institute of Cancer and Genomic Sciences, Center for Computational Biology, University of Birmingham, UK; c Institute of Translational Medicine, University Hospitals Birmingham Nhs, Foundation Trust, UK; d Nihr Surgical Reconstruction and Microbiology Research Center, University Hospital Birmingham, Birmingham, UK; e Department of Gastroenterology, University Hospitals Birmingham Nhs Foundation Trust, Birmingham, UK; f Institute of Cancer and Genomic Sciences, University of Birmingham, Birmingham, UK; g University of Birmingham Microbiome Treatment Center, University of Birmingham, UK; h Center for Liver and Gastroenterology Research, Nihr Birmingham Biomedical Research Center, University of Birmingham, Birmingham, UK; i Institute of Applied Health Research, University of Birmingham, UK; j Department of Clinical Chemistry, Vu University Medical Center, Amsterdam, The Netherlands; k Spaarne Gasthuis, Department of Gastroenterology and Hepatology, Spaarne Gasthuis (primary institute), Hoofddorp and Haarlem, The Netherlands; l Olvg West, Department of Gastroenterology and Hepatology, Onze Lieve Vrouwe Gasthuis West, Amsterdam, The Netherlands; m Medical Research Counsil, MRC Health Data Research, UK; n NIHR Experimental Cancer Medicine Center, National Institute for Health Research, Birmingham, UK; o NIHR Biomedical Research Center, University Hospital Birmingham, Birmingham, UK; p Amsterdam Umc, Vu University Amsterdam, Department of Paediatric Gastroenterology, Ag&m Research Institute, Amsterdam, The Netherlands

**Keywords:** Omics, biomarker, surveillance, adenoma, colorectal cancer

## Abstract

The risk of recurrent dysplastic colonic lesions is increased following polypectomy. Yield of endoscopic surveillance after adenoma removal is low, while interval colorectal cancers occur. To longitudinally assess the dynamics of fecal microbiota and amino acids in the presence of adenomatous lesions and after their endoscopic removal. In this longitudinal case–control study, patients collected fecal samples prior to bowel preparation before scheduled colonoscopy and 3 months after this intervention. Based on colonoscopy outcomes, patients with advanced adenomas and nonadvanced adenomas (0.5–1.0 cm) who underwent polypectomy during endoscopy (*n* = 19) were strictly matched on age, body-mass index, and smoking habits to controls without endoscopic abnormalities (*n* = 19). Microbial taxa were measured by 16S RNA sequencing, and amino acids (AA) were measured by high-performance liquid chromatography (HPLC). Adenoma patients were discriminated from controls based on AA and microbial composition. Levels of proline (*p* = .001), ornithine (*p* = .02) and serine (*p* = .02) were increased in adenoma patients compared to controls but decreased to resemble those of controls after adenoma removal. These AAs were combined as a potential adenoma-specific panel (AUC 0.79(0.64–0.94)). For bacterial taxa, differences between patients with adenomas and controls were found (*Bifidobacterium* spp.↓, *Anaerostipes* spp.↓, *Butyricimonas* spp.↑, *Faecalitalea* spp.↑ *and Catenibacterium* spp.↑), but no alterations in relative abundance were observed after polypectomy. Furthermore, *Faecalitalea* spp. and *Butyricimonas* spp. were significantly correlated with adenoma-specific amino acids. We selected an amino acid panel specifically increased in the presence of adenomas and a microbial signature present in adenoma patients, irrespective of polypectomy. Upon validation, these panels may improve the effectiveness of the surveillance program by detection of high-risk individuals and determination of surveillance endoscopy timing, leading to less unnecessary endoscopies and less interval cancer.

## Introduction

Colorectal cancer (CRC) is among the three malignancies with the highest incidence and mortality rate worldwide.^1^ Precancerous dysplastic polyps or advanced adenomas may develop from benign polyps after undergoing a sequence of mutations over a period of decades. The majority of CRC originates from this so-called adenoma-carcinoma sequence.^2^ CRC-related mortality has decreased significantly over the past years due to population-based screening programs, in which early detection and removal of neoplastic (precursor) lesions are established.^3^ In the US, the CRC screening program is mainly performed by 10-yearly colonoscopy in which all adenomas are required to be removed, whereas in Europe, guidelines recommend a more cost-effective approach, using fecal immunochemical tests (FIT) to select high-risk individuals for endoscopic screening.^4,5^ However, sensitivity of FIT is limited; advanced adenomas are missed in 43–61% of the cases and for nonadvanced adenomas, this percentage is even higher.^6^ This underlines the need to improve screening strategies.

Furthermore, after the first screening, endoscopic surveillance is recommended at set intervals dependent on the characteristics of removed adenomas, as the risk of recurrent dysplastic lesions remains increased in these patients.^7^ These intervals have, however, been defined only by expert opinion and the yield of pathology is low while interval cancer still occurs (for example, 1.8% CRC yield vs 0.6% interval cancers).^8,9^ No noninvasive markers have yet been validated to improve the timing of the surveillance endoscopies.

There is growing evidence that dysbiosis of the gut microbiota results in changes in human and microbial metabolism in the gut, leading to a change in metabolic end products, amongst which amino acids.^10^ The gut microbiota is involved in colonic metabolism during the progression of advanced adenoma into CRC.^11^ Several human studies have identified unique microbial signatures in the presence of adenomas, such as the presence of *Fusobacterium nucleatum* and cyclomodulin-positive *Escherichia coli*, which are thought to play a role in colonic inflammation and tumorigenesis.^12–15^ The increased inflammatory state as well as carcinogenesis and alteration in microbial abundances lead to the excretion of metabolic end products, including amino acids (AA). Specific AA have previously been associated with inflammation and tumorigenesis (e.g. proline, leucine and ethanolamine).^16–19^ This suggests that both gut bacteria and AA may hold potential as biomarkers for the identification of high-risk individuals for adenomas and CRC.

The aim of this study was to investigate the potential of microbiota and AA profiles for the identification and surveillance of adenoma patients. In addition, we aimed to gain insight into the interplay between microbiota and metabolism before and after adenoma removal.

## Results

### Clinical data

A total of 32 patients with a successful polypectomy and 32 controls agreed with participation and collected two subsequent samples prior to and 3 months after colonoscopy. Of these participants, we were able to strictly match 19 polypectomy patients (advanced adenomas and nonadvanced adenomas combined) to 19 controls on age, BMI and smoking habits. From the adenoma group, a total of 9 patients had advanced adenomas and 10 patients had non-advanced adenoma (NA), with a median size of the largest adenoma being 0.7 cm [IQR 0.5–0.8]. Baseline demographics are depicted in Table 1. Based on Mann–Whitney U and Chi square tests, there were no significant differences in age, gender BMI and smoking habits between groups.

**Table 1.** Demographics

	Polypectomy (n = 19)	Controls (n = 19)
Age (mean, ±SD)	73 ± 6.1	68 ± 10.4
Gender (n, males, %)	17 89.5]	13 [68.4]
BMI (mean, ±SD)	26.8 ± 8.3	25.6 ± 3.6
Smoking status (n, %)		
Active	2 [10.5]	2 [10.5]
Quit	13 [68.4]	11 [57.9]
Never	4 [21.1]	6 [31.6]
Indication for endoscopic assessment (n, %)		
Positive FIT	6 [31.6]	3 [15.8]
Rectal blood loss	3 [15.8]	1 [5.2]
Change in bowel habits	2 [10.5]	2 [10.5]
Surveillance after polypectomy	2 [10.5]	2 [10.5]
Abdominal Pain	1 [5.2]	6 [31.6]
Diarrhea	1 [5.2]	0 [0]
Anemia	0 [0]	1 [5.2]
Incontinence	1 [5.2]	0 [0]
Family history CRC	1 [5.2]	1 [5.2]
Surveillance after CRC surgery	1 [5.2]	1 [5.2]
Other	1 [5.2]	2 []
ABx 3 months prior to inclusion	1 [5.2]	4 [21.1]
ABx 3 months prior to second sample	0 [0]	1 [5.2]
Size adenoma cm (mean, ±SD)	0.9 ± 0.6	NA
Localization of adenoma (n, %)*		
Cecum	3 [15.8]	NA
Colon Ascendens	4 [21.1]	NA
Flexura Hepatica	0 [0]	NA
Colon Transversum	7 [36.8]	NA
Flexura Lienalis	1 [5.2]	NA
Colon Descendens	0 [0]	NA
Sigmoid	7 [36.8]	NA
Rectum	3 [15.8]	NA
Adenoma characteristics (largest adenoma) (n, [%])		
≥ 10 mm	8 [42.1]	NA
Villous histology	4 [21.1]	NA
HGD	0 [0]	NA
No dysplasia	0 [0]	NA
Hyperplasia	0 [0]	NA
LGD	19 [100]	NA
Sessile/serrated	0 [0]	NA
Total number adenomas removed (n, [%])		
1	5 [26.3]	NA
2	6 [31.6]	NA
3	6 [31.6]	NA
4	1 [5.2]	NA
8	1 [5.2]	NA

Baseline characteristics of participants in a polypectomy follow-up study. Abbreviations: BMI, body-mass index; ABx: antibiotics. Based on Mann–Whitney U and Chi square tests, there were no significant differences in age, gender BMI and smoking habits between groups. * 1 value missing.

### Amino acid analysis

**Figure 1.**
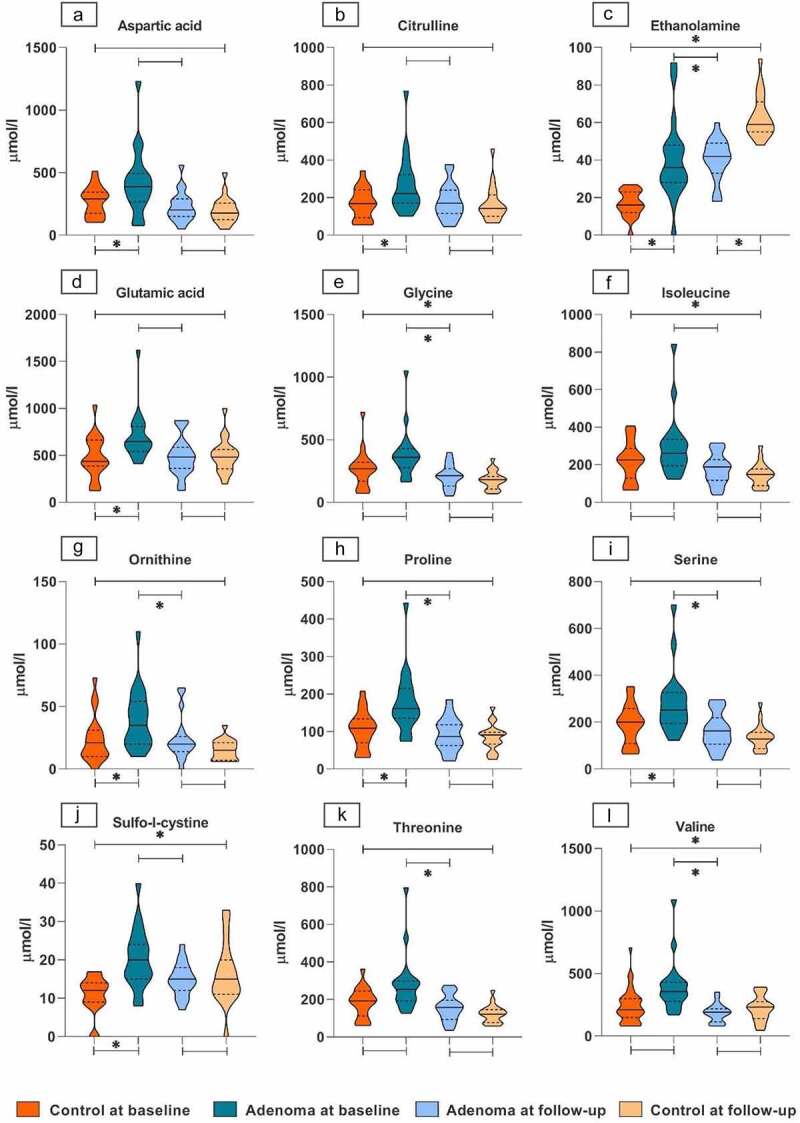
Distribution of amino acids among all study groups.

A total of 42 different amino acids were obtained from the high-performance liquidchromatography (HPLC) analysis, of which 21 were excluded due to undetectable or unquantifiable levels and 21 were eligible for statistical analysis. Distribution within subgroups is visualized for all selected amino acids as shown in Figure 1.

Violin plots for the distribution of absolute amino acid levels per group. Depicted are the all amino acids meeting to the criteria of significance in at least one comparison of this study, meaning a selection frequency >50 during stability analysis based on Elastic Net selection and a *p*-value<0.05 in univariate analysis. The Y-axis in every plot depicts the absolute value of the corresponding amino acid, the X-axis depicts the study groups. Control groups are depicted in Orange and adenoma groups in blue. Dark colors correspond with baseline measurements and light colors with follow-up. The violin plots are depicted per group per amino acid. The horizontal line within the violin plots represents the median, and the dotted lines represent the upper- and lower quartiles. Broadness of the violin plot depicts the density of samples at the corresponding amino acid levels. Significance is indicated by a star above of under the lines connecting study groups.

### Adenoma patients versus controls prior to endoscopy

Based on our machine learning pipeline, eight amino acids met the criteria of statistical significance. These were sulfo-l-cysteine, ethanolamine, proline, ornithine, citrulline, serine, aspartic acid and glutamic acid, of which levels were all increased in adenoma patients (Figure 1). Histidine was selected by Elastic Net (EN) analysis but was not significant in univariate analysis. The corresponding frequencies and *p*-values of significant amino acids are given in Supplementary table 1.

### Adenoma patients versus controls post-treatment

When comparing adenoma patients post-polypectomy to controls post-endoscopy, only ethanolamine levels remained significantly different and increased in the samples of adenoma patients (Figure 1).

### Adenoma patients pre- versus post-polypectomy

Seven unique amino acids differed significantly between fecal samples of adenoma patients prior to and 3 months after polypectomy. Selected amino acids were ethanolamine, threonine, serine, proline, valine, glycine and ornithine. Ethanolamine was increased after adenoma removal, whereas the latter six were decreased (Figure 1). The corresponding frequencies and *p*-values are reported in Supplementary table 1.

### Controls pre- versus post-endoscopy

Ethanolamine, valine, sulfo-l-cystine, isoleucine and glycine were considered significantly different between controls pre- and post-endoscopy, of which the first three were increased after endoscopy and the latter two were decreased (Supplementary Table 1, Figure 1). For ethanolamine, glycine and valine this same behavior was seen in adenoma patients pre- and post-polypectomy.

### Microbial composition

**Figure 2.**
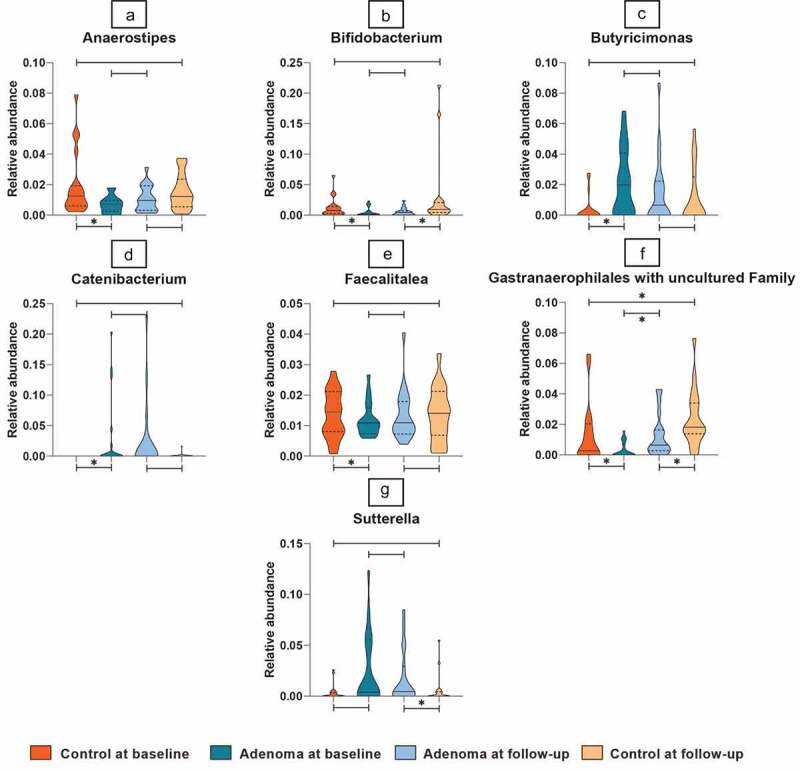
Distribution of selected taxa for adenomas versus controls at baseline and follow-up.

**Figure 3.**
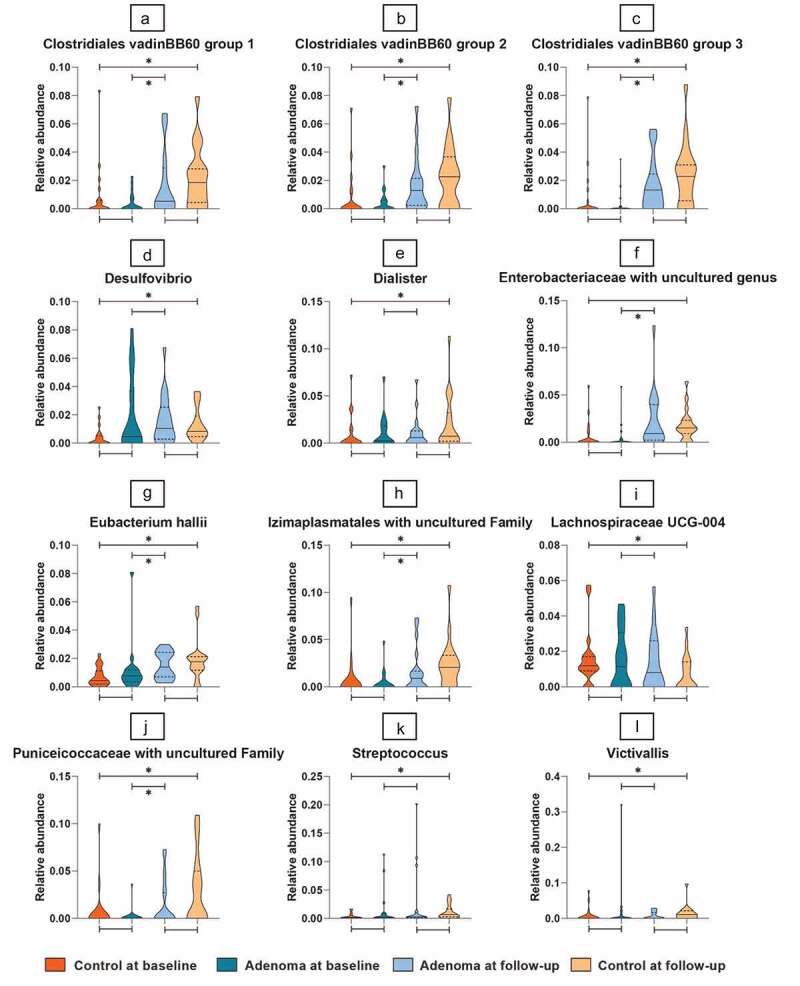
Distribution of selected taxa for baseline versus follow-up.

In our present study, 2,246,463 high-quality reads were obtained with a median count of 23.041 reads per sample. After taxonomic assignment, 211 operational taxonomic units (OTUs) were obtained throughout the samples (Supplementary Table 2). No significant differences were seen in alpha and beta diversity between any of the comparisons. The proportions of the dominant taxa were assessed at the phylum level both at the baseline and follow-up and are depicted in bar plots in Figures 2–3.

The distribution of taxa selected to differentiate between adenomas and controls across all four subgroups. Depicted are the microbial taxa meeting the criteria of significance of a Linear Discriminant Analysis (LDA) value >2. On the *Y*-axis, the relative abundance per taxa is presented in violin plots. On the *X*-axis, study groups are given. Dark Orange depicts control samples at baseline, dark blue depicts adenomas at baseline, light blue depicts adenomas at follow-up, and light-Orange depicts controls at follow-up. The horizontal line within the violin plots represents the median and the dotted lines represent the upper- and lower quartiles. Broadness of the violin plot depicts the density of samples at the corresponding taxa relative abundancy. Significance is indicated by a star above of under the lines connecting study groups.

The distribution of taxa selected to differentiate between baseline and follow-up across all four subgroups. Depicted are the microbial taxa meeting the criteria of significance of a Linear Discriminant Analysis (LDA) value >2. On the Y-axis, the relative abundance per taxa is presented in violin plots. On the *X*-axis, study groups are given. Dark Orange depicts control samples at baseline, dark blue depicts adenomas at baseline, light blue depicts adenomas at follow-up, light-orange depicts controls at follow-up. The horizontal line within the violin plots represents the median and the dotted lines represent the upper and lower quartiles. Broadness of the violin plot depicts the density of samples at the corresponding taxa relative abundancy. Significance is indicated by a star above of under the lines connecting study groups.

### Adenoma versus controls prior to endoscopy

Prior to endoscopic intervention, the relative abundances of six taxa were significantly different between fecal samples of adenoma patients and controls. Relative abundances of three taxa increased in adenoma patients: *Butyricimonas*spp.,*Catenibacterium* spp. *and Faecalitalea* spp. and the abundance of three taxa was decreased in adenoma patients compared to controls: *Anaerostipes* spp.,*Bifidobacterium* spp. and Cyanobacteria within the *Gastranaerophilales* order with uncultured family. An overview of these taxa is given in Supplementary table 1. Distribution of these taxa is depicted in Figure 2.

### Adenoma patients versus controls post-treatment

When comparing adenoma patients 3 months after polypectomy to controls 3 months after endoscopy, the relative abundance of the three taxa that were increased in the adenoma group at the baseline was no longer significantly different from controls. Furthermore, two out of three taxa decreased in the adenoma group at the baseline (*Bifidobacterium* and Cyanobacteria within the *Gastranaerophilales* order with uncultured family) consistently remained increased at follow-up when compared to controls. The taxa *Sutterella* was also increased in adenoma compared to control samples at follow-up. The behavior of these selected species is depicted for all subgroups in Figure 2.

### Adenoma patients pre- versus post-polypectomy

A total of eight taxa were identified as significantly different between adenoma samples at the baseline and 3 months after polypectomy and relative abundances were all increased at the second measurement. These were Cyanobacteria within the *Gastranaerophilales* order with uncultured family, Firmicutes within three of the *Clostridiales vadinBB60 groups* with uncultured genuses, *Eubacterium hallii*, Proteobacteria within the *Enterobacteriaceae* family with uncultured genus, Verrucomicrobiae from the *Puniceicoccaceae* family with uncultured genus, Tenericutes within the *Izimaplasmatales* order with an uncultured family (Supplementary table 2, Figure 3).

### Controls pre- versus post-endoscopy

When comparing control samples pre- versus post-endoscopy, the same taxa that differentiated adenoma patients at the baseline and at follow-up were significant, plus an additional five new taxa, highlighting a large effect of endoscopy on fecal microbiota. One of these new taxa, *Lachnospiraceae UCG4* spp., was increased in the baseline samples. The other four, *Streptococcus* spp.,*Dialister* spp.,*Desulfovibrio* spp. and *Victivallis* spp., were increased in the follow-up samples of controls. An overview of the significant taxa is given in Supplementary table 1 for both comparisons. Distribution of these taxa across all subgroups are visualized in Figure 3.

### Testing of adenoma-specific panel for screening and monitoring purposes

For bacterial taxa, only *Gastranaerophilales* with uncultured family was significantly different in both the comparison between adenomas and controls at the baseline and between adenomas pre- and post-polypectomy. However, as its relative abundance differed between all four subgroups, this was not considered potentially adenoma-specific. Based on amino acid analysis, four amino acids were selected both in the comparison between adenomas and controls and between adenomas pre- and post-polypectomy. These were ethanolamine, proline, ornithine and serine. As ethanolamine was significantly different for all study comparisons, this amino acid was excluded as a potential adenoma-specific marker. Proline, ornithine and serine were combined as potential adenoma-specific panels. Based on logistic regression with a 10-fold cross validation, we obtained an AUC value of 0.84 (95%CI 0.81–89) for the training set and an AUC of 0.79 (0.64–0.94) for the test set. The corresponding sensitivity and specificity are given in Supplementary table 3.

### Biological links between microbial taxa and amino acids

Based on Pearson's correlation, links were observed between microbial taxa and amino acids differentiating patients with adenoma from controls. The *Faecalitalea* genus was positively correlated with serine (*r* = 0.45; *p* = .005), citrulline (*r* = 0.43; *p* = .008), glutamic acid (*r* = 0.39; *p* = .02) and proline (*r* = 0.38; *p* = .02). The *Butyricimonas* genus was correlated with serine (*r* = 0.31; *p* = .056), citrulline (*r* = 0.35; *p* = .03) and glutamic acid (*r* = 0.35; *p* = .03). A supplementary figure is added to understand the data distribution and correlation.

## Discussion

In the present prospective case–control study, we assessed adenoma-associated gut microbiota and fecal amino acid levels both prior to and after endoscopic removal. Patients with adenomas and controls could be discriminated prior to intervention based on both microbial taxa and amino acid profiles. Amino acids returned to the normal state after polypectomy. None of the microbial taxa differentiating patients with adenoma from controls changed significantly in adenoma patients after polypectomy was performed.

It is not yet elucidated whether the microbial signatures of CRC patients are a cause or consequence of the adenoma-carcinoma sequence. As levels of adenoma-specific taxa did not change significantly after adenoma removal, the microbial adenoma signature seems to be of limited potential as surveillance biomarkers to identify adenoma growth. In the current study, we did reveal specific taxonomic abundances associated with adenoma patients, irrespective of resection. Some microbial taxa were increased in patients with adenoma compared to controls. These included the genus *Faecalitalea* and *Butyricimonas*, of which positive correlations were found with some of the adenoma-specific amino acids (i.e. proline and serine). When assessing metabolic pathways in the online available KEGG database, multiple genes from these taxa correspond with enzymes in the metabolic pathways of serine, proline, glutamate and ornithine. Interestingly, as further discussed below, upregulated proline and serine metabolism have been observed in patients with CRC.^13,17,20^ Based on these findings, it may be hypothesized that microbial taxa from the genera *Faecalitalea* and *Butyricimonas* are linked to carcinogenesis. In addition, relative abundances of other taxa were decreased in adenoma patients. For example, *Bifidobacterium* was significantly decreased in adenoma patients compared to controls both before and after adenoma removal. This same phenomenon, though only partly significant, was observed for *Catenibacterium* and *Sutterella*, of which relative abundances were increased in all adenoma samples. In line with this, previous studies have reported a decrease of *Bifidobacteria* abundance in the samples of CRC patients.^12,13^ It is believed that this taxa is important for the maintenance of bacterial diversity and intestinal homeostasis, and as such, a dysbiosis of this taxa is thought to promote CRC development.^21^ In addition, both *Catenibacterium* (family Erysipelotrichidae) and *Sutterella* (family Betaproteobacteria) have previously been described as upregulated in the adenoma-carcinoma sequence.^22–24^ In the current study, we observed that these taxonomic differences remained present after adenoma removal. The stability of microbial abundance in adenoma patients, irrespective of removal, was also described in a previous study investigating the fecal microbiota of adenoma patients pre- and post-treatment.^25^ These findings suggest that a lack or overgrowth of these specific microbial taxa may drive tumorigenesis. Should these taxa indeed drive the adenoma-carcinoma sequence, removal of present adenomas would solely decrease the short-term risk of developing CRC while maintaining the underlying carcinogenic environment. In this case, it would be valuable to identify these high-risk individuals, allowing for a personalized, targeted surveillance.

Furthermore, it is important to note that the influence of endoscopic intervention on microbial taxa was larger than polypectomy itself, as all taxa with different abundances between adenoma patients pre- and post-polypectomy were similar to those differentiating controls pre- and post-endoscopy. Whether this has any health-related consequences cannot be answered based on this study; however, these findings are important both when conducting research and when performing microbiota-driven diagnostic testing as bowel lavage and endoscopy should be considered an important cause of bias.

Contrary to the microbiota profiles, several adenoma-specific amino acids were detected in the current study. Proline, ornithine and serine differentiated between adenomas and controls at the baseline and between adenomas pre- and post-polypectomy. These amino acids more closely resembled the control group after adenoma removal. The observation that fecal amino acids levels returned to the normal state upon adenoma removal substantiates their potential to serve as a noninvasive biomarker panel for adenoma detection and also to determine the timing of surveillance endoscopy. In previous studies, elevated levels of these ‘adenoma-specific’ amino’acid have been presented in patients with colorectal cancer.^11,13,26^ Proline, an amino acid released during cell stress, can be metabolized to produce ATP and reactive oxygen species (ROS) or may lead to apoptosis or autophagy. This amino acid has consistently been presented as a contributor to tumor cell survival.^16,17^ In addition, serine is thought to play a key role in tumor growth as this amino acid is an important precursor for the biosynthesis of many molecules. An increase in intracellular serine biosynthesis from glucose has been reported in cancer cells repeatedly.^20^ Increased serum levels of ornithine in CRC patients have also been reported previously as a decreased consumption is associated with colorectal carcinogenesis.^27,28^ The above literature findings may explain the increased concentration of proline, serine and ornithine in fecal samples of patients with (large) adenomas, as they may reflect early malignant neoplastic changes in metabolism. Another pathophysiological explanation for the altered amino acid composition in adenoma patients may be disease-specific changes in gut microbiota, which may lead to alteration of amino acid metabolism. As mentioned above, increased relative abundances of some of the microbial taxa including *Faecalitalea* and *Butyricimonas* were present in patients with adenomas compared to controls, whereas abundances more closely resembled controls after adenoma removal. Even though no significant differences in their abundances were seen when comparing pre- and post- polypectomy samples, positive correlations were found between these taxa and some of the adenoma-specific amino acids (i.e. proline and serine). Therefore, it could be hypothesized that amino acid alterations may either be a result or consequence of specific changes in the gut microbiota composition.

We observed remarkable behavior of ethanolamine. This AA has been described to play a role in carcinogenesis and tumor progression and has therefore been presented as a potential cancer screening biomarker.^18,29^ In line with this, upregulation of ethanolamine was observed in adenoma as compared to controls. However, in follow-up samples, ethanolamine was increased both in adenoma patients post-polypectomy and in controls after endoscopy. Since ethanolamine plays an important role in epithelial proliferation, it is likely that the bowel lavage with laxatives or the endoscopic intervention itself affects the fecal ethanolamine levels more profoundly than the presence of premalignant lesions. Thus, its potential for timing of surveillance seems to be limited.^30,31^

Currently, no noninvasive markers are available for timing of surveillance, leading to undesirable high variation between protocols. Surveillance of adenoma patients has been the subject of a previous study comparing stool hemoglobin levels (FIT), with colonoscopy outcomes of 5225 participants 1 yafter polypectomy.^32^ These tests were characterized by a sensitivity of 27.6–51.7% and 17.0–33.0% for CRC and advanced adenomas, respectively, depending on the applied cutoff values. Replacing colonoscopy with FIT would reduce the number of colonoscopies by approximately 70% but would lead to an unacceptable high number of missed CRC and advanced adenoma cases.^6,33^ Here, we presented accuracy rates of adenoma-specific amino acid profiles, which surpass the performance of the FIT. This underlines the potential of this noninvasive stool test to be applied in clinical practice.

To the best of our knowledge, this was the first study in which both fecal microbiota and AA composition were assessed in a cohort of adenoma patients undergoing polypectomy. Strengths include the prospective design in which all patients were classified according to endoscopic findings, interventions and pathology reports, which is currently the gold standard for the detection of colonic abnormalities. In addition, this protocol ensured for a group of control patients without any colonic abnormalities and allowed for a reliable comparison at follow-up, as potential influences of endoscopy and bowel preparation on microbiota and metabolomics outcome were taken into account. To limit the risk of selection bias, cases and controls were strictly matched on age, BMI and smoking habits, which are known factors associated with changes in microbial composition.^34^ The limitation of this study was the small number of inclusions, which may have led to false-negative results and limited the possibility of a subgroup analysis between low- and high-risk adenomas. With respect to the data on microbiota composition, this may mean that some taxa may have been adenoma specific but did not reach statistical significance due to the limited number of included patients. Furthermore, even though amino acids have previously been presented increased in patients with CRC, we were not able to validate the adenoma-specific amino acid panel in an external validation set as there was no online data available from previous studies using similar research groups and techniques. Our current findings on the AUC values may therefore be an overestimation of the accuracy in the screening and surveillance population. Still, as no noninvasive markers are available yet, we do believe that following validation, these new AA and microbiota panels may have additional value for future adenoma surveillance. In the future, the currently presented adenoma-specific AA panel and high-risk microbial signature should be validated in a new prospective dataset. Upon validation, these new biomarker panels should be tested separately and in combination with the current polypectomy surveillance program. In addition, fecal AA profiles should be tested for their robustness under different environmental circumstances before further development into screening tests should be pursued.

In conclusion, we longitudinally assessed the adenoma-associated gut microbiota and fecal amino acid expression, prior to and after polypectomy. We defined a microbial signature that may allow for stratification of high-risk individuals. Furthermore, we presented a panel of amino acids increased in the presence of adenomas and returning to normal following removal. Upon validation, this panel may serve as a noninvasive marker for adenomas and allow for the development of a scientifically based protocol on timing of surveillance endoscopy, improving effectiveness and decreasing incidence of interval cancer.

## Patients and methods

### Study design

This multicenter prospective case–control study was performed as part of a larger study on CRC biomarker discovery including 1039 patients between May 2017 and November 2017 in one tertiary referral hospital (Amsterdam UMC, location VUmc) and two district hospitals (OLVG West, Amsterdam and Spaarne Gasthuis, Hoofddorp and Haarlem), all located in The Netherlands. This study was approved on 04–09–2014 by the Medical Ethical Review Committee (METc) of Amsterdam UMC (2014.404) and by local METcs of OLVG West and Spaarne Gasthuis. Written informed consent was obtained from all participants.

### Study participants and sample collection

A workflow similar to a previous study was adopted.^35^ In short, patients >18 years of age with a scheduled colonoscopy at one of the three hospitals were asked to participate in this study, irrespective of endoscopy indication. Based on endoscopic and histological findings, patients were divided into different groups. For the current study, patients were eligible to take part in the case of (a) presence of advanced adenoma(s) with successful polypectomy during endoscopy, (b) presence of nonadvanced adenoma(s) (NA) with successful polypectomy during endoscopy, (c) controls, that is, patients with no abnormalities observed during endoscopy (excluding small anal fibroma, hemorrhoids and/or diverticula), no interventions performed during endoscopy, and, in the case of mucosal biopsies, no histological abnormalities. The advanced adenoma group was characterized by polyps ≥1 cm in diameter, or with villous histology, or high-grade dysplasia, according to the European Society of Gastrointestinal Endoscopy (ESGE) guidelines.^36^ The NA group was defined as adenomas without villous histology or high-grade dysplasia, sized 0.5–1.0 cm in diameter. Patients who underwent a polypectomy were matched to controls on age, BMI and smoking habits in a 1:1 ratio. Exclusion criteria for both subgroups of this study were the presence of an underlying gastrointestinal disease (such as inflammatory bowel disease, CRC, celiac disease), incomplete endoscopic assessment due to various reasons (e.g. hampered visibility due to inadequate bowel cleansing and incomplete colonoscopy due to pain), inability to collect or store sufficient fecal sample mass to perform analyses, incomplete removal of polyps for the polypectomy group and inability to match a case to a control participant based on strict predefined criteria.

All patients collected one fecal sample (Stuhlgefäß 10 ml, Frickenhausen, Germany) in the week prior to bowel preparation. These samples were stored in their own freezer at home within 1 hour following bowel movement and brought to the hospital on the day of their endoscopic assessment. Directly upon arrival at the hospital, the samples were stored at −24°C. In addition, all participants were asked to complete a questionnaire, which included items on demographic characteristics such as age, gender, body mass index (BMI) and smoking habits. Questions on comorbidity and medication use were included. Participants eligible for the current study were asked to collect a second fecal sample 3 months after their endoscopy and to complete a second questionnaire (following the same procedure as the first sample and questionnaire).

### Endoscopic and histologic evaluation

All endoscopies were either performed or supervised by trained gastroenterologists. Using the electronic patient files, reports from endoscopy and histology outcome of mucosal biopsies and/or polypectomy were obtained. The endoscopy reports were used as the standard reference for both localization and total number of removed adenomas in this study. The histology reports were used as reference for size, differentiation grade (e.g. hyperplasia, dysplasia) and villous aspects. The presence of sessile and/or serrated characteristics was noted for all nonadvanced adenomas. In the case where multiple polyps were present and removed, this classification was based on the most advanced/largest lesion.

### Sample preparation

Frozen subsamples of 500 mg per participant were divided for both AA and microbiota analysis while remaining on dry ice. Subsamples were weighed on a calibrated scale and transferred into glass vials (20 ml headspace vial, Thames Restek, Saunderton, UK). The subsamples were then transported on dry ice to the designated laboratories. Samples for amino acid measurements were brought to the metabolic laboratory of the clinical chemistry department at the Amsterdam UMC, location VUmc. The samples for microbiota analysis were transported to the Institute of Immunology and Immunotherapy of the University of Birmingham (UK).

### Targeted amino acid analysis

By means of the standard operating procedure that have previously been published, fecal samples were analyzed using HPLC.^37^ In short, the 500 mg fecal subsample was mixed by vortex with 1000 µL of distilled water for 1 minto homogenize the samples. The samples were investigated by a laboratory researcher (ES) who was blinded for the diagnosis. Samples were first frozen at −30° and then freeze-dried for 24 h (Christ Alpha 2–4), to avoid bias by differences in fecal consistency. The residual after freeze-drying was mixed with distilled water, consistently maintaining a feces–water ratio of 100 mg:5 mL. Before the analysis of amino acids, this mixture was again vigorously homogenized. A total of 400 µL of the mixture was pipetted into a filter and centrifuged for 20 min at 14.000*g* (Hettig Zentrifugen Mikro 2 R). In a one-to-one ratio, the supernatant was mixed with an internal standard solution before centrifuging for 10 min. The final mixture was then filtered (Whatman) into compatible containers for the final amino acid analyses (Biochrome 30). Amino acids were separated by ion-exchange chromatography and detected by UV-absorbance after post-column derivatization with ninhydrin. As per our protocol, amino acids were excluded from further analysis if levels were unquantifiable or undetectable in at least one of the study subjects per group to circumvent possible over- or underestimation of the results. Levels of 5 μmol or lower were considered unquantifiable and levels of 0 μmol/l were considered undetectable.

### Microbial 16S rRNA profiling

Extracted paired DNA was used for 16S rRNA gene amplification and sequencing, as part of the Qiagen AllPrep DNA/RNA Mini Kit, strictly following the Earth Microbiome Project

protocol.^38^ Using primer targeting, the 16s rRNA V4 region (515 F-806 R) in a one-step, single-indexed PCR approach, the 16s rRNA genes were amplified in duplicate. This was done in batches, using the appropriate negative controls. Subsequently, paired-end sequencing (2x250bp) was performed on an Illumina MiSeq platform (Illumina, Dan Diego, US) and processed the pipeline Quantitative Insights Into Microbial Ecology 2 (QIIME2).^39^ Taxonomy was assigned against the Silva-132–99% OTU database.^40^

### Statistical analyses

Both AA profiles and microbiota data were normalized using autoscaling (mean-centered and divided by SD of each variable). For data on microbial profiles, relative abundances per study group were analyzed using linear discriminant analysis (LDA) effect size (LEfSe).^41^ Taxa with LDA>2 and a *p*-value below 0.05 were considered significant. For data on amino acids, univariate analysis was performed on each amino acid based on Mann–Whitney U tests for the unpaired data and based on the Wilcoxon signed rank tests for the paired data. Multivariate analyses were performed using Elastic Net (EN) analysis.^42^ This is a variable selection method extended from the linear regression method. It automatically selects the best subset of features linked with the outcome variable. EN uses a penalty parameter, λ (range: 0 to 1) that provides a sparse solution. Penalty parameters are optimized using 10-fold cross validation. The stronger the penalty (close to 1), the smaller the number of selected variables, while the weaker the penalty (close to 0) higher numbers of variables are selected based on EN analysis. The penalty is applied to the sum of the absolute values of the regression coefficients (L1 norm) and then L2 (Ridge penalty) is applied. The L1 penalty encourages the sparse representation, whereas L2 stabilizes the solution.^42^ This method has an improved performance when the number of features are significantly larger than the number of samples, by allowing for grouped selection or the selection of correlated variables.^43,44^ We applied a generalized linear model (GLM) on the variables identified by EN to cater for the stability analysis of the selected features.^43,44^ The process was repeated 100 times and the features were ranked according to their respective selection frequency associated with each run. Amino acids were considered significantly different between groups when selected by EN analysis (defined as a selection frequency >50) combined with a *p*-value <0.05 in the univariate analysis. Then, amino acids and bacterial taxa that met the significance criteria for the comparison between adenomas and controls at baseline, as well as for the comparisons between adenomas pre- and post-polypectomy were considered as adenoma-specific markers. Combination of this biomarker panel was used in logistic regression with a 10-fold cross validation to generate performance matrices with confidence intervals, sensitivity and specificity-values. Two AUCs were generated, one on the training data set, and another on the 10-fold random sampling treated as test set. In addition, we performed Pearson correlation and estimated correlation coefficient (r) to determine associations between the selected amino acids and microbial taxa across adenoma and control samples.

## Supplementary Material

Supplemental Material

## Data Availability

Upon acceptance, raw data will be made publicly available via the DOI registration. Data can currently be accessed via the following link: https://figshare.com/s/d7b177c77c17c0c4ada4

## Supplementary material

Supplemental data for this article can be accessed on the publisher’s https://figshare.com/s/19c5eef9bbb8f6afecd8

## References

[1] Ferlay J, Parkin DM, Steliarova-Foucher E. Estimates of cancer incidence and mortality in Europe in 2008. Eur J Cancer. 2010;46(4):765–15. doi:10.1016/j.ejca.2009.12.014. 20116997

[2] Ballinger AB, Anggiansah C. Colorectal cancer. BMJ. 2007;335(7622):715–718. doi:10.1136/bmj.39321.527384.BE. 17916855 PMC2001051

[3] Zorzi M, Fedeli U, Schievano E, Bovo E, Guzzinati S, Baracco S, Fedato C, Saugo M, Dei Tos AP. Impact on colorectal cancer mortality of screening programmes based on the faecal immunochemical test. Gut. 2015;64(5):784–790. doi:10.1136/gutjnl-2014-307508. 25179811

[4] Levin B, Lieberman, DA, McFarland B, Bond J, Dash C, Giardiello FM, Glick S, Johnson D, Johnson CD, Levin TR, Pickhardt PJ, Rex DK, Smith RA, Thorson A, Winawer SJ, et al. Screening and surveillance for the early detection of colorectal cancer and adenomatous polyps, 2008: a joint guideline from the American cancer society, the US Multi-society task force on colorectal cancer, and the American college of radiology. Gastroenterol. 2008;134(5):1570–1595. doi:10.1053/j.gastro.2008.02.002. 18384785

[5] Hoff G, Dominitz JA. Contrasting US and European approaches to colorectal cancer screening: which is best? Gut. 2010;59(3):407–414. doi:10.1136/gut.2009.192948. 20207645

[6] Katsoula A, Paschos P, Haidich A-B, Tsapas A, Giouleme O. Diagnostic accuracy of fecal immunochemical test in patients at increased risk for colorectal cancer: a meta-analysis. JAMA Intern Med. 2017;177(8):1110–1118. doi:10.1001/jamainternmed.2017.2309. 28628706 PMC5710432

[7] Hassan C, Quintero E, Dumonceau J-M, Regula J, Brandão C, Chaussade S, Dekker E, Dinis-Ribeiro M, Ferlitsch M, Gimeno-García A, et al. Post-polypectomy colonoscopy surveillance: European society of gastrointestinal endoscopy (ESGE) guideline. Endoscopy. 2013;45(10):842–851. doi:10.1055/s-0033-1344548. 24030244

[8] Atkin W, et al. Adenoma surveillance and colorectal cancer incidence: a retrospective, multicentre, cohort study. Lancet Oncol. 2017;18(6):823–834. doi:10.1016/S1470-2045(17)30187-0. 28457708 PMC5461371

[9] Robertson DJ, Lieberman DA, Winawer SJ, Ahnen DJ, Baron JA, Schatzkin A, Cross AJ, Zauber AG, Church TR, Lance P, et al. Colorectal cancers soon after colonoscopy: a pooled multicohort analysis. Gut. 2014;63(6):949–956. doi:10.1136/gutjnl-2012-303796. 23793224 PMC4383397

[10] Heinken A, Hertel J, Thiele I. Metabolic modelling reveals broad changes in gut microbial metabolism in inflammatory bowel disease patients with dysbiosis. NPJ Syst Biol Appl. 2021;7(1):19. doi:10.1038/s41540-021-00178-6. 33958598 PMC8102608

[11] Weir TL, Manter DK, Sheflin AM, Barnett BA, Heuberger AL, Ryan EP, et al. Stool microbiome and metabolome differences between colorectal cancer patients and healthy adults. PLoS One. 2013;8(8):e70803. doi:10.1371/journal.pone.0070803. 23940645 PMC3735522

[12] Thomas AM, Manghi P, Asnicar F, Pasolli E, Armanini F, Zolfo M, Beghini F, Manara S, Karcher N, Pozzi C, et al. Metagenomic analysis of colorectal cancer datasets identifies cross-cohort microbial diagnostic signatures and a link with choline degradation. Nat Med. 2019;25(4):667–678. doi:10.1038/s41591-019-0405-7. 30936548 PMC9533319

[13] Yachida S, Mizutani S, Shiroma H, Shiba S, Nakajima T, Sakamoto T, Watanabe H, Masuda K, Nishimoto Y, Kubo M, et al. Metagenomic and metabolomic analyses reveal distinct stage-specific phenotypes of the gut microbiota in colorectal cancer. Nat Med. 2019;25(6):968–976. doi:10.1038/s41591-019-0458-7. 31171880

[14] Yang Y, Weng W, Peng J, Hong L, Yang L, Toiyama Y, Gao R, Liu M, Yin M, Pan C, et al. Fusobacterium nucleatum increases proliferation of colorectal cancer cells and tumor development in mice by activating toll-like receptor 4 signaling to nuclear factor-kappaB, and up-regulating expression of MicroRNA-21. Gastroenterol. 2017;152(4):851–866 e24. doi:10.1053/j.gastro.2016.11.018. PMC5555435 27876571

[15] Bonnet M, Buc E, Sauvanet P, Darcha C, Dubois D, Pereira B, Déchelotte P, Bonnet R, Pezet D, Darfeuille-Michaud A, et al. Colonization of the human gut by E. coli and colorectal cancer risk. Clin Cancer Res. 2014;20(4):859–867. doi:10.1158/1078-0432.CCR-13-1343. 24334760

[16] Olivares O, Mayers JR, Gouirand V, Torrence ME, Gicquel T, Borge L, Lac S, Roques J, Lavaut M-N, Berthezène P, et al. Collagen-derived proline promotes pancreatic ductal adenocarcinoma cell survival under nutrient limited conditions. Nat Commun. 2017;8(1):16031. doi:10.1038/ncomms16031. 28685754 PMC5504351

[17] Guo L, Cui C, Zhang K, Wang J, Wang Y, Lu Y, Chen K, Yuan J, Xiang G, Tang B, Sun Y, Wu C, et al. Kindlin-2 links mechano-environment to proline synthesis and tumor growth. Nat Commun. 2019;10(1):845. doi:10.1038/s41467-019-08772-3. 30783087 PMC6381112

[18] Cheng M, Bhujwalla ZM, Glunde K. Targeting phospholipid metabolism in cancer. Front Oncol. 2016;6:266. doi:10.3389/fonc.2016.00266. 28083512 PMC5187387

[19] Salisbury TB, Arthur S. The regulation and function of the L-type amino acid transporter 1 (LAT1) in Cancer. Int J Mol Sci. 2018;19(8):2373. doi:10.3390/ijms19082373. PMC6121554 30103560

[20] Mattaini KR, Sullivan MR, Vander Heiden MG. The importance of serine metabolism in cancer. J Cell Biol. 2016;214(3):249–257. doi:10.1083/jcb.201604085. 27458133 PMC4970329

[21] O’Callaghan A, van Sinderen D. Bifidobacteria and their role as members of the human gut microbiota. Front Microbiol. 2016;7:925. doi:10.3389/fmicb.2016.00925. 27379055 PMC4908950

[22] Wirbel J, Pyl PT, Kartal E, Zych K, Kashani A, Milanese A, Fleck JS, Voigt AY, Palleja A, Ponnudurai R, et al. Meta-analysis of fecal metagenomes reveals global microbial signatures that are specific for colorectal cancer. Nat Med. 2019;25(4):679–689. doi:10.1038/s41591-019-0406-6. 30936547 PMC7984229

[23] Sarhadi V, Lahti L, Saberi F, Youssef O, Kokkola A, Karla T, Tikkanen M, Rautelin H, Puolakkainen P, Salehi R, Knuutila S, et al. Gut microbiota and host gene mutations in colorectal cancer patients and controls of Iranian and Finnish origin. Anticancer Res. 2020;40(3):1325–1334. doi:10.21873/anticanres.14074. 32132029

[24] Mori G, Rampelli S, Orena BS, Rengucci C, De Maio G, Barbieri G, Passardi A, Gardini AC, Frassineti GL, Gaiarsa S, Albertini AM, Ranzani GN, Calistri D, Pasca MR, et al. Shifts of faecal microbiota during sporadic colorectal carcinogenesis. Sci Rep. 2018;8(1):10329. doi:10.1038/s41598-018-28671-9. 29985435 PMC6037773

[25] Sze MA, Baxter NT, Ruffin MT, Rogers MAM, Schloss PD. Normalization of the microbiota in patients after treatment for colonic lesions. Microbiome. 2017;5(1):150. doi:10.1186/s40168-017-0366-3. 29145893 PMC5689185

[26] Wang X, Wang J, Rao B, Deng L, et al. Gut flora profiling and fecal metabolite composition of colorectal cancer patients and healthy individuals. Exp Ther Med. 2017;13(6):2848–2854. doi:10.3892/etm.2017.4367. 28587349 PMC5450625

[27] Hashim NAA, Ab-Rahim S, Suddin LS, Saman MSA, Mazlan M. Global serum metabolomics profiling of colorectal cancer. Mol Clin Oncol. 2019;11(1):3–14. doi:10.3892/mco.2019.1853. 31289671 PMC6535638

[28] Wei Z, Song J, Wang G, Cui X, Zheng J, Tang Y, Chen X, Li J, Cui L, Liu C-Y, et al. Deacetylation of serine hydroxymethyl-transferase 2 by SIRT3 promotes colorectal carcinogenesis. Nat Commun. 2018;9(1):4468. doi:10.1038/s41467-018-06812-y. 30367038 PMC6203763

[29] Ormsby MJ, Logan M, Johnson SA, McIntosh A, Fallata G, Papadopoulou R, Papachristou E, Hold GL, Hansen R, Ijaz UZ, et al. Inflammation associated ethanolamine facilitates infection by Crohn’s disease-linked adherent-invasive *Escherichia coli*. EBioMedicine. 2019;43:325–332. doi:10.1016/j.ebiom.2019.03.071. 31036531 PMC6557746

[30] Yang H, Xiong X, Li T, Yin Y. Ethanolamine enhances the proliferation of intestinal epithelial cells via the mTOR signaling pathway and mitochondrial function. Vitro Cell Dev Biol Anim. 2016;52(5):562–567. doi:10.1007/s11626-016-0002-8. 27083163

[31] Zhou J, Xiong X, Wang K, Zou L, Lv D, Yin Y, et al. Ethanolamine metabolism in the mammalian gastrointestinal tract: mechanisms, patterns, and importance. Curr Mol Med. 2017;17(2):92–99. doi:10.2174/1566524017666170331161715. 28429671

[32] Cross AJ, Wooldrage K, Robbins EC, Kralj-Hans I, MacRae E, Piggott C, Stenson I, Prendergast A, Patel B, Pack K, et al. Faecal immunochemical tests (FIT) versus colonoscopy for surveillance after screening and polypectomy: a diagnostic accuracy and cost-effectiveness study. Gut. 2019;68(9):1642–1652. doi:10.1136/gutjnl-2018-317297. 30538097 PMC6709777

[33] Rozen P, Levi Z, Hazazi R, Waked A, Vilkin A, Moaz E, Birkenfeld S, Leshno M, Niv Y, et al. Identification of colorectal adenomas by a quantitative immunochemical faecal occult blood screening test depends on adenoma characteristics, development threshold used and number of tests performed. Aliment Pharmacol Ther. 2009;29(8):906–917. doi:10.1111/j.1365-2036.2009.03946.x. 19183147

[34] Kobayashi T, Jin J-S, Kibe R, Touyama M, Tanaka Y, Benno Y, Fujiwara K, Shimakawa M, Maruo T, Toda T, et al. Identification of human intestinal microbiota of 92 men by data mining for 5 characteristics, i.e., age, BMI, smoking habit, cessation period of previous smokers and drinking habit. Biosci Microbiota Food Health. 2013;32(4):129–137. doi:10.12938/bmfh.32.129. 24936372 PMC4034333

[35] Bosch S, Bot R, Wicaksono A, Savelkoul E, van Der Hulst R, Kuijvenhoven J, Stokker P, Daulton E, Covington JA, de Meij TGJ, de Boer NKH, et al. Early detection and follow-up of colorectal neoplasia based on faecal volatile organic compounds. Colorectal Dis. 2020;22(9):1119–1129. doi:10.1111/codi.15009. 32040880

[36] Brenner H, Hoffmeister M, Stegmaier C, Brenner G, Altenhofen L, Haug U. Risk of progression of advanced adenomas to colorectal cancer by age and sex: estimates based on 840,149 screening colonoscopies. Gut. 2007;56(11):1585–1589. doi:10.1136/gut.2007.122739. 17591622 PMC2095643

[37] Bosch S, Struys EA, van Gaal N, Bakkali A, Jansen EEW, Diederen K, Benninga MA, Mulder CJJ, de Boer NKH, de Meij TGJ, et al. Fecal amino acid analysis can discriminate de novo treatment-naive pediatric inflammatory bowel disease from controls. J Pediatr Gastroenterol Nutr. 2018 May;66(5):773-778. Doi:10.1097/MPG.0000000000001812. 29112087

[38] Project EM. 16S illumina amplication protocol.

[39] Bolyen E, Ram Rideout J, Chase J, Anders Pitman T, Shiffer A, Mercurio W, R Dillon M, Gregory Caporaso J. An introduction to applied bioinformatics: a free, open, and interactive text. J Open Source Educ. 2018;1(5):27. doi:10.21105/jose.00027. 30687845 PMC6343836

[40] Quast C, Pruesse E, Yilmaz P, Gerken J, Schweer T, Yarza P, Peplies J, Glöckner FO. The SILVA ribosomal RNA gene database project: improved data processing and web-based tools. Nucleic Acids Res. 2013;41(Database issue):D590–6. doi:10.1093/nar/gks1219. 23193283 PMC3531112

[41] Segata N, Izard J, Waldron L, Gevers D, Miropolsky L, Garrett WS, Huttenhower C. Metagenomic biomarker discovery and explanation. Genome Biol. 2011;12(6):R60. doi:10.1186/gb-2011-12-6-r60. 21702898 PMC3218848

[42] Hui Zou TH, Hastie T. Regularization and variable selection via the elastic net. R Stat Soc. 2005;67(2):230–301. doi:10.1111/j.1467-9868.2005.00503.x.

[43] Bravo-Merodio L, Acharjee A, Hazeldine J, Bentley C, Foster M, Gkoutos GV, Lord JM. Machine learning for the detection of early immunological markers as predictors of multi-organ dysfunction. Sci Data. 2019;6(1):328. doi:10.1038/s41597-019-0337-6. 31857590 PMC6923383

[44] Bravo-Merodio L, Williams JA, Gkoutos GV, Acharjee A. -Omics biomarker identification pipeline for translational medicine. J Transl Med. 2019;17(1):155. doi:10.1186/s12967-019-1912-5. 31088492 PMC6518609

