# Supplementary material for: The potential of fecal microbiota and amino acids to detect and monitor patients with adenoma
Source: Gut Microbes. 2022 Feb 21;14(1):2038863. doi: 10.1080/19490976.2022.2038863 (PMC8865277; doi:10.1080/19490976.2022.2038863)
Supplement: Supplemental Material [file KGMI_A_2038863_SM4476.zip › supplementary/downloadFromZipFile.pdf]

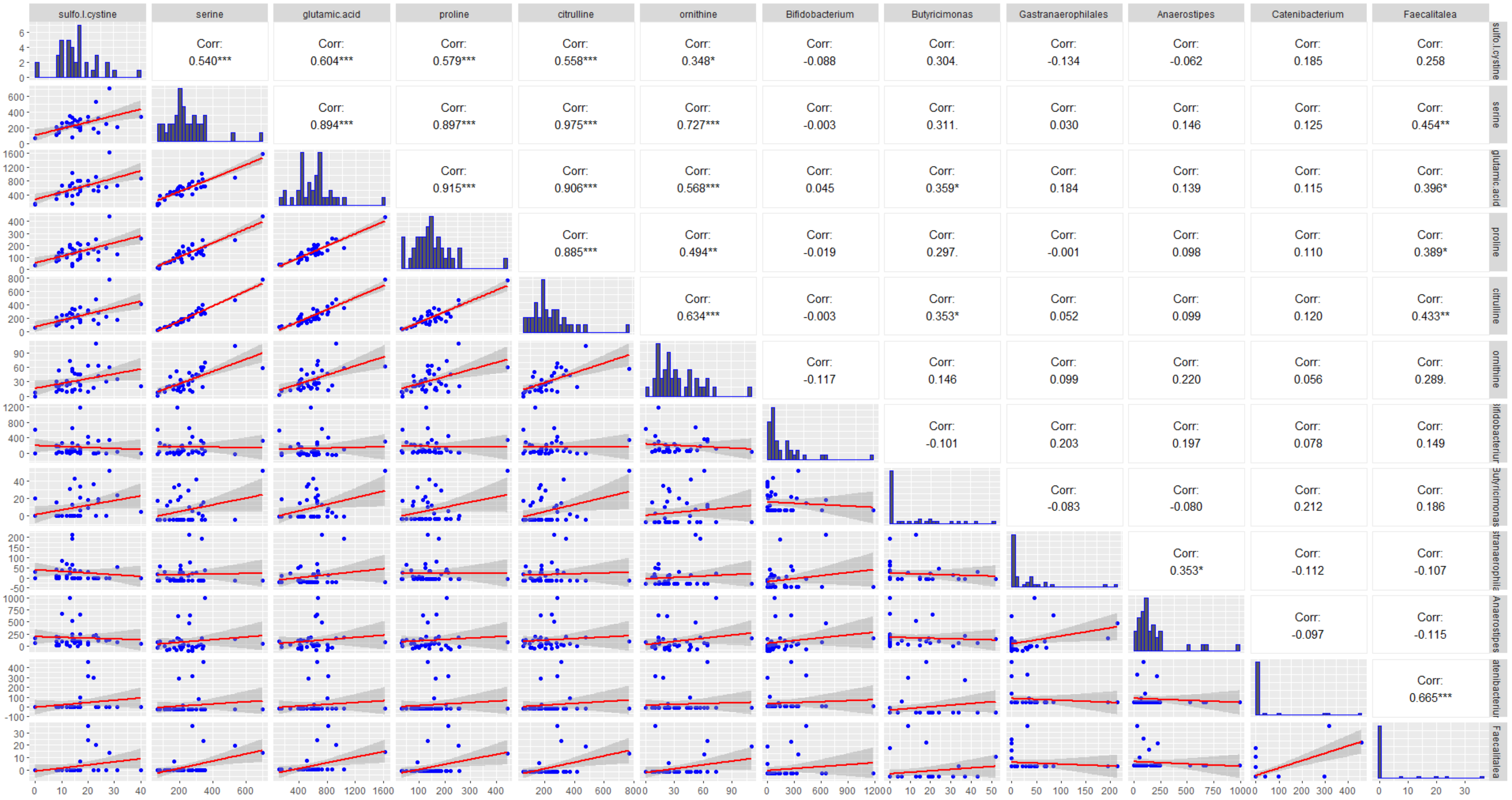

Supplementary Figure 1. Correlation coefficients are given for all selected amino acids and microbial taxa. The diagonal mid-line provides distribution of all amino acids. "*" = p-value is < 0.001; "**" = p-value is < 0.01; "*" = p-value is < 0.05; "."= p-value is < 0.10**
