# Supplementary material for: The potential of fecal microbiota and amino acids to detect and monitor patients with adenoma
Source: Gut Microbes. 2022 Feb 21;14(1):2038863. doi: 10.1080/19490976.2022.2038863 (PMC8865277; doi:10.1080/19490976.2022.2038863)
Supplement: Supplemental Material [file KGMI_A_2038863_SM4476.zip › supplementary/Supplementaly table 1.docx]

**Supplementary table 1. Overview of selected markers from amino acid and microbiota database for all four comparisons with corresponding effect size, frequency and *p-*values.**

| **Adenoma versus controls at baseline** | | |
| --- | --- | --- |
| **Microbiota** | | |
| *Butyricimonas* spp*.* | | |
| *Catenibacterium* spp*.* | | |
| *Faecalitalea* spp*.* | | |
| *Gastranaerophilales* with uncultured family | | |
| *Anaerostipes* spp*.* | | |
| *Bifidobacterium* spp*.* | | |
| **Amino acids** | Frequency | *p*-value |
| Ethanolamine | 100 | 0,000001 |
| Sulfo.l.cystine | 100 | 0,00005 |
| Proline | 85 | 0,0010 |
| Ornithine | 76 | 0,0200 |
| Citrulline | 60 | 0,0200 |
| Serine | 57 | 0,0200 |
| Aspartic acid | 53 | 0,0300 |
| Glutamic acid | 53 | 0,0080 |
| **Adenoma samples and control at follow-up** | | |
| **Microbiota** | |  |
| *Bifidobacterium* spp*.* | | |
| *Gastranaerophilales* with uncultured family | | |
| *Sutterella* spp. | | |
| **Amino acids** | Frequency | *p*-value |
| Ethanolamine | 100 | 2,87E-08 |
| **Adenoma at baseline and post-polypectomy** | | |
| **Microbiota** | | |
| Clostridiales vadinBB60 group 2 | | |
| *Eubacterium* hallii | | |
| Enterobacteriaceae with uncultured genus | | |
| *Puniceicoccaceae* with uncultured family | | |
| Clostridiales vadinBB60 group | | |
| *Izimaplasmatales* with uncultured family | | |
| Clostridiales vadinBB60 group 1 | | |
| *Gastranaerophilales* with uncultured family | | |
| **Amino acids** | Frequency | *p*-value |
| Ethanolamine | 100 | 0,000396 |
| Glycine | 100 | 0,000132 |
| Ornitine | 100 | 0,000231 |
| Threonine | 100 | 0,000132 |
| Valine | 99 | 0,000132 |
| Proline | 97 | 0,000155 |
| Serine | 97 | 0,000132 |
| **Control samples at baseline and post-endoscopy** | | |
| **Microbiota** | | |
| *Lachnospiraceae UCG004* spp. | | |
| *Clostridiales vadinBB60 group 2* | | |
| *Eubacterium* hallii | | |
| Enterobacteriaceae with uncultured genus | | |
| *Streptococcus* spp. | | |
| *Dialister* spp. | | |
| *Puniceicoccaceae* with uncultured family | | |
| *Clostridiales vadinBB60 group* | | |
| Izimaplasmatales with uncultured family | | |
| *Clostridiales vadinBB60 group 1* | | |
| *Gastranaerophilales* with uncultured family | | |
| Desulfovibrio | | |
| Victivallis | | |
| **Amino acids** | Frequency | *p*-value |
| Ethanolamine | 100 | 0,00013 |
| Sulfo-l-cystine | 75 | 0,001 |
| Valine | 73 | 0,003 |
| Glycine | 63 | 0,005 |
| Isoleucine | 61 | 0,004 |

Supplementaly table 1. Overview of selected markers for all four comparisons. All microbial taxa were selected because of an LDA>2 and p-value < 0.05. Abbreviations: LDA = linear discriminant component. Frequencies are based on Elastic Net (EN) selection after stability analysis based on 100 repetitions. Corresponding p-values are based on the Mann-Whitney U test for unpaired samples and on Wilcoxon signed rank tests for paired samples. In this study, LDA>2 was considered significant for microbial taxa. A frequency above 50 combined with a -value below 0.05 was considered significant for amino acids.
