# Supplementary material for: The potential of fecal microbiota and amino acids to detect and monitor patients with adenoma
Source: Gut Microbes. 2022 Feb 21;14(1):2038863. doi: 10.1080/19490976.2022.2038863 (PMC8865277; doi:10.1080/19490976.2022.2038863)
Supplement: Supplemental Material [file KGMI_A_2038863_SM4476.zip › supplementary/Supplementary Table 2 OTUs.docx]

| **OTU** | **Kingdom** | **Phylum** | **Class** | **Order** | **Family** | **Genus** |
| --- | --- | --- | --- | --- | --- | --- |
| OTU1 | Archaea | Euryarchaeota | Methanobacteria | Methanobacteriales | Methanobacteriaceae | Methanobrevibacter |
| OTU2 | Archaea | Euryarchaeota | Methanobacteria | Methanobacteriales | Methanobacteriaceae | Methanosphaera |
| OTU3 | Archaea | Euryarchaeota | Thermoplasmata | Methanomassiliicoccales | Methanomassiliicoccaceae | Methanomassiliicoccus |
| OTU4 | Bacteria | Actinobacteria | Actinobacteria | Actinomycetales | Actinomycetaceae | Actinomyces |
| OTU5 | Bacteria | Actinobacteria | Actinobacteria | Bifidobacteriales | Bifidobacteriaceae | Bifidobacterium |
| OTU6 | Bacteria | Actinobacteria | Actinobacteria | Bifidobacteriales | Bifidobacteriaceae |  |
| OTU7 | Bacteria | Actinobacteria | Coriobacteriia | Coriobacteriales | Atopobiaceae | Libanicoccus |
| OTU8 | Bacteria | Actinobacteria | Coriobacteriia | Coriobacteriales | Atopobiaceae | Olsenella |
| OTU9 | Bacteria | Actinobacteria | Coriobacteriia | Coriobacteriales | Coriobacteriaceae | Collinsella |
| OTU10 | Bacteria | Actinobacteria | Coriobacteriia | Coriobacteriales | Coriobacteriales Incertae Sedis | uncultured |
| OTU11 | Bacteria | Actinobacteria | Coriobacteriia | Coriobacteriales | Eggerthellaceae | Adlercreutzia |
| OTU12 | Bacteria | Actinobacteria | Coriobacteriia | Coriobacteriales | Eggerthellaceae | Enterorhabdus |
| OTU13 | Bacteria | Actinobacteria | Coriobacteriia | Coriobacteriales | Eggerthellaceae | Senegalimassilia |
| OTU14 | Bacteria | Actinobacteria | Coriobacteriia | Coriobacteriales | Eggerthellaceae | Slackia |
| OTU15 | Bacteria | Actinobacteria | Coriobacteriia | Coriobacteriales | Eggerthellaceae | uncultured |
| OTU16 | Bacteria | Actinobacteria | Coriobacteriia | Coriobacteriales | Eggerthellaceae |  |
| OTU17 | Bacteria | Actinobacteria | Coriobacteriia | Coriobacteriales | uncultured | uncultured bacterium |
| OTU18 | Bacteria | Bacteroidetes | Bacteroidia | Bacteroidales | Bacteroidaceae | Bacteroides |
| OTU19 | Bacteria | Bacteroidetes | Bacteroidia | Bacteroidales | Barnesiellaceae | Barnesiella |
| OTU20 | Bacteria | Bacteroidetes | Bacteroidia | Bacteroidales | Barnesiellaceae | Coprobacter |
| OTU21 | Bacteria | Bacteroidetes | Bacteroidia | Bacteroidales | Barnesiellaceae | uncultured |
| OTU22 | Bacteria | Bacteroidetes | Bacteroidia | Bacteroidales | Marinifilaceae | Butyricimonas |
| OTU23 | Bacteria | Bacteroidetes | Bacteroidia | Bacteroidales | Marinifilaceae | Odoribacter |
| OTU24 | Bacteria | Bacteroidetes | Bacteroidia | Bacteroidales | Marinifilaceae | Sanguibacteroides |
| OTU25 | Bacteria | Bacteroidetes | Bacteroidia | Bacteroidales | Muribaculaceae | uncultured Porphyromonadaceae bacterium |
| OTU26 | Bacteria | Bacteroidetes | Bacteroidia | Bacteroidales | Muribaculaceae | uncultured bacterium |
| OTU27 | Bacteria | Bacteroidetes | Bacteroidia | Bacteroidales | Muribaculaceae |  |
| OTU28 | Bacteria | Bacteroidetes | Bacteroidia | Bacteroidales | Porphyromonadaceae | Porphyromonas |
| OTU29 | Bacteria | Bacteroidetes | Bacteroidia | Bacteroidales | Prevotellaceae | Alloprevotella |
| OTU30 | Bacteria | Bacteroidetes | Bacteroidia | Bacteroidales | Prevotellaceae | Paraprevotella |
| OTU31 | Bacteria | Bacteroidetes | Bacteroidia | Bacteroidales | Prevotellaceae | Prevotella |
| OTU32 | Bacteria | Bacteroidetes | Bacteroidia | Bacteroidales | Prevotellaceae | Prevotella 2 |
| OTU33 | Bacteria | Bacteroidetes | Bacteroidia | Bacteroidales | Prevotellaceae | Prevotella 7 |
| OTU34 | Bacteria | Bacteroidetes | Bacteroidia | Bacteroidales | Prevotellaceae | Prevotella 9 |
| OTU35 | Bacteria | Bacteroidetes | Bacteroidia | Bacteroidales | Prevotellaceae | Prevotellaceae NK3B31 group |
| OTU36 | Bacteria | Bacteroidetes | Bacteroidia | Bacteroidales | Prevotellaceae | Prevotellaceae UCG-001 |
| OTU37 | Bacteria | Bacteroidetes | Bacteroidia | Bacteroidales | Prevotellaceae | uncultured |
| OTU38 | Bacteria | Bacteroidetes | Bacteroidia | Bacteroidales | Rikenellaceae | Alistipes |
| OTU39 | Bacteria | Bacteroidetes | Bacteroidia | Bacteroidales | Rikenellaceae | Rikenellaceae RC9 gut group |
| OTU40 | Bacteria | Bacteroidetes | Bacteroidia | Bacteroidales | Tannerellaceae | Parabacteroides |
| OTU41 | Bacteria | Bacteroidetes | Bacteroidia | Bacteroidales | uncultured | gut metagenome |
| OTU42 | Bacteria | Bacteroidetes | Bacteroidia | Bacteroidales | uncultured | uncultured bacterium |
| OTU43 | Bacteria | Bacteroidetes | Bacteroidia | Flavobacteriales | Flavobacteriaceae | uncultured |
| OTU44 | Bacteria | Cyanobacteria | Melainabacteria | Gastranaerophilales | uncultured bacterium | uncultured bacterium |
| OTU45 | Bacteria | Cyanobacteria | Melainabacteria | Gastranaerophilales | uncultured rumen bacterium | uncultured rumen bacterium |
| OTU46 | Bacteria | Cyanobacteria | Oxyphotobacteria | Chloroplast |  |  |
| OTU47 | Bacteria | Firmicutes | Bacilli | Bacillales | Family XI | Gemella |
| OTU48 | Bacteria | Firmicutes | Bacilli | Lactobacillales | Enterococcaceae | Enterococcus |
| OTU49 | Bacteria | Firmicutes | Bacilli | Lactobacillales | Lactobacillaceae | Lactobacillus |
| OTU50 | Bacteria | Firmicutes | Bacilli | Lactobacillales | Leuconostocaceae | Leuconostoc |
| OTU51 | Bacteria | Firmicutes | Bacilli | Lactobacillales | Leuconostocaceae | Weissella |
| OTU52 | Bacteria | Firmicutes | Bacilli | Lactobacillales | Streptococcaceae | Lactococcus |
| OTU53 | Bacteria | Firmicutes | Bacilli | Lactobacillales | Streptococcaceae | Streptococcus |
| OTU54 | Bacteria | Firmicutes | Clostridia | Clostridiales | Christensenellaceae | Christensenellaceae R-7 group |
| OTU55 | Bacteria | Firmicutes | Clostridia | Clostridiales | Christensenellaceae | uncultured |
| OTU56 | Bacteria | Firmicutes | Clostridia | Clostridiales | Christensenellaceae |  |
| OTU57 | Bacteria | Firmicutes | Clostridia | Clostridiales | Clostridiaceae 1 | Clostridium sensu stricto 1 |
| OTU58 | Bacteria | Firmicutes | Clostridia | Clostridiales | Clostridiales vadinBB60 group | uncultured bacterium |
| OTU59 | Bacteria | Firmicutes | Clostridia | Clostridiales | Clostridiales vadinBB60 group | uncultured organism |
| OTU60 | Bacteria | Firmicutes | Clostridia | Clostridiales | Clostridiales vadinBB60 group |  |
| OTU61 | Bacteria | Firmicutes | Clostridia | Clostridiales | Defluviitaleaceae | Defluviitaleaceae UCG-011 |
| OTU62 | Bacteria | Firmicutes | Clostridia | Clostridiales | Eubacteriaceae | Anaerofustis |
| OTU63 | Bacteria | Firmicutes | Clostridia | Clostridiales | Eubacteriaceae | Eubacterium |
| OTU64 | Bacteria | Firmicutes | Clostridia | Clostridiales | Family XIII | Family XIII AD3011 group |
| OTU65 | Bacteria | Firmicutes | Clostridia | Clostridiales | Family XIII | Family XIII UCG-001 |
| OTU66 | Bacteria | Firmicutes | Clostridia | Clostridiales | Family XIII | Mogibacterium |
| OTU67 | Bacteria | Firmicutes | Clostridia | Clostridiales | Family XIII | [Eubacterium] brachy group |
| OTU68 | Bacteria | Firmicutes | Clostridia | Clostridiales | Family XIII | [Eubacterium] nodatum group |
| OTU69 | Bacteria | Firmicutes | Clostridia | Clostridiales | Family XIII |  |
| OTU70 | Bacteria | Firmicutes | Clostridia | Clostridiales | Lachnospiraceae | Agathobacter |
| OTU71 | Bacteria | Firmicutes | Clostridia | Clostridiales | Lachnospiraceae | Anaerostipes |
| OTU72 | Bacteria | Firmicutes | Clostridia | Clostridiales | Lachnospiraceae | Blautia |
| OTU73 | Bacteria | Firmicutes | Clostridia | Clostridiales | Lachnospiraceae | Butyrivibrio |
| OTU74 | Bacteria | Firmicutes | Clostridia | Clostridiales | Lachnospiraceae | CAG-56 |
| OTU75 | Bacteria | Firmicutes | Clostridia | Clostridiales | Lachnospiraceae | Coprococcus 1 |
| OTU76 | Bacteria | Firmicutes | Clostridia | Clostridiales | Lachnospiraceae | Coprococcus 2 |
| OTU77 | Bacteria | Firmicutes | Clostridia | Clostridiales | Lachnospiraceae | Coprococcus 3 |
| OTU78 | Bacteria | Firmicutes | Clostridia | Clostridiales | Lachnospiraceae | Dorea |
| OTU79 | Bacteria | Firmicutes | Clostridia | Clostridiales | Lachnospiraceae | Eisenbergiella |
| OTU80 | Bacteria | Firmicutes | Clostridia | Clostridiales | Lachnospiraceae | Fusicatenibacter |
| OTU81 | Bacteria | Firmicutes | Clostridia | Clostridiales | Lachnospiraceae | GCA-900066575 |
| OTU82 | Bacteria | Firmicutes | Clostridia | Clostridiales | Lachnospiraceae | Howardella |
| OTU83 | Bacteria | Firmicutes | Clostridia | Clostridiales | Lachnospiraceae | Hungatella |
| OTU84 | Bacteria | Firmicutes | Clostridia | Clostridiales | Lachnospiraceae | Lachnoclostridium |
| OTU85 | Bacteria | Firmicutes | Clostridia | Clostridiales | Lachnospiraceae | Lachnospira |
| OTU86 | Bacteria | Firmicutes | Clostridia | Clostridiales | Lachnospiraceae | Lachnospiraceae AC2044 group |
| OTU87 | Bacteria | Firmicutes | Clostridia | Clostridiales | Lachnospiraceae | Lachnospiraceae FCS020 group |
| OTU88 | Bacteria | Firmicutes | Clostridia | Clostridiales | Lachnospiraceae | Lachnospiraceae ND3007 group |
| OTU89 | Bacteria | Firmicutes | Clostridia | Clostridiales | Lachnospiraceae | Lachnospiraceae NK4A136 group |
| OTU90 | Bacteria | Firmicutes | Clostridia | Clostridiales | Lachnospiraceae | Lachnospiraceae NK4B4 group |
| OTU91 | Bacteria | Firmicutes | Clostridia | Clostridiales | Lachnospiraceae | Lachnospiraceae UCG-001 |
| OTU92 | Bacteria | Firmicutes | Clostridia | Clostridiales | Lachnospiraceae | Lachnospiraceae UCG-003 |
| OTU93 | Bacteria | Firmicutes | Clostridia | Clostridiales | Lachnospiraceae | Lachnospiraceae UCG-004 |
| OTU94 | Bacteria | Firmicutes | Clostridia | Clostridiales | Lachnospiraceae | Marvinbryantia |
| OTU95 | Bacteria | Firmicutes | Clostridia | Clostridiales | Lachnospiraceae | Oribacterium |
| OTU96 | Bacteria | Firmicutes | Clostridia | Clostridiales | Lachnospiraceae | Roseburia |
| OTU97 | Bacteria | Firmicutes | Clostridia | Clostridiales | Lachnospiraceae | Sellimonas |
| OTU98 | Bacteria | Firmicutes | Clostridia | Clostridiales | Lachnospiraceae | Shuttleworthia |
| OTU99 | Bacteria | Firmicutes | Clostridia | Clostridiales | Lachnospiraceae | Syntrophococcus |
| OTU100 | Bacteria | Firmicutes | Clostridia | Clostridiales | Lachnospiraceae | Tyzzerella |
| OTU101 | Bacteria | Firmicutes | Clostridia | Clostridiales | Lachnospiraceae | Tyzzerella 3 |
| OTU102 | Bacteria | Firmicutes | Clostridia | Clostridiales | Lachnospiraceae | Tyzzerella 4 |
| OTU103 | Bacteria | Firmicutes | Clostridia | Clostridiales | Lachnospiraceae | [Bacteroides] pectinophilus group |
| OTU104 | Bacteria | Firmicutes | Clostridia | Clostridiales | Lachnospiraceae | [Eubacterium] eligens group |
| OTU105 | Bacteria | Firmicutes | Clostridia | Clostridiales | Lachnospiraceae | [Eubacterium] fissicatena group |
| OTU106 | Bacteria | Firmicutes | Clostridia | Clostridiales | Lachnospiraceae | [Eubacterium] hallii group |
| OTU107 | Bacteria | Firmicutes | Clostridia | Clostridiales | Lachnospiraceae | [Eubacterium] ruminantium group |
| OTU108 | Bacteria | Firmicutes | Clostridia | Clostridiales | Lachnospiraceae | [Eubacterium] ventriosum group |
| OTU109 | Bacteria | Firmicutes | Clostridia | Clostridiales | Lachnospiraceae | [Eubacterium] xylanophilum group |
| OTU110 | Bacteria | Firmicutes | Clostridia | Clostridiales | Lachnospiraceae | [Ruminococcus] gauvreauii group |
| OTU111 | Bacteria | Firmicutes | Clostridia | Clostridiales | Lachnospiraceae | [Ruminococcus] gnavus group |
| OTU112 | Bacteria | Firmicutes | Clostridia | Clostridiales | Lachnospiraceae | [Ruminococcus] torques group |
| OTU113 | Bacteria | Firmicutes | Clostridia | Clostridiales | Lachnospiraceae | uncultured |
| OTU114 | Bacteria | Firmicutes | Clostridia | Clostridiales | Lachnospiraceae |  |
| OTU115 | Bacteria | Firmicutes | Clostridia | Clostridiales | Peptococcaceae | Peptococcus |
| OTU116 | Bacteria | Firmicutes | Clostridia | Clostridiales | Peptococcaceae | uncultured |
| OTU117 | Bacteria | Firmicutes | Clostridia | Clostridiales | Peptostreptococcaceae | Intestinibacter |
| OTU118 | Bacteria | Firmicutes | Clostridia | Clostridiales | Peptostreptococcaceae | Romboutsia |
| OTU119 | Bacteria | Firmicutes | Clostridia | Clostridiales | Peptostreptococcaceae | Terrisporobacter |
| OTU120 | Bacteria | Firmicutes | Clostridia | Clostridiales | Ruminococcaceae | Anaerotruncus |
| OTU121 | Bacteria | Firmicutes | Clostridia | Clostridiales | Ruminococcaceae | Butyricicoccus |
| OTU122 | Bacteria | Firmicutes | Clostridia | Clostridiales | Ruminococcaceae | CAG-352 |
| OTU123 | Bacteria | Firmicutes | Clostridia | Clostridiales | Ruminococcaceae | Candidatus Soleaferrea |
| OTU124 | Bacteria | Firmicutes | Clostridia | Clostridiales | Ruminococcaceae | Caproiciproducens |
| OTU125 | Bacteria | Firmicutes | Clostridia | Clostridiales | Ruminococcaceae | DTU089 |
| OTU126 | Bacteria | Firmicutes | Clostridia | Clostridiales | Ruminococcaceae | Faecalibacterium |
| OTU127 | Bacteria | Firmicutes | Clostridia | Clostridiales | Ruminococcaceae | Flavonifractor |
| OTU128 | Bacteria | Firmicutes | Clostridia | Clostridiales | Ruminococcaceae | Fournierella |
| OTU129 | Bacteria | Firmicutes | Clostridia | Clostridiales | Ruminococcaceae | GCA-900066225 |
| OTU130 | Bacteria | Firmicutes | Clostridia | Clostridiales | Ruminococcaceae | Hydrogenoanaerobacterium |
| OTU131 | Bacteria | Firmicutes | Clostridia | Clostridiales | Ruminococcaceae | Negativibacillus |
| OTU132 | Bacteria | Firmicutes | Clostridia | Clostridiales | Ruminococcaceae | Oscillibacter |
| OTU133 | Bacteria | Firmicutes | Clostridia | Clostridiales | Ruminococcaceae | Oscillospira |
| OTU134 | Bacteria | Firmicutes | Clostridia | Clostridiales | Ruminococcaceae | Ruminiclostridium 1 |
| OTU135 | Bacteria | Firmicutes | Clostridia | Clostridiales | Ruminococcaceae | Ruminiclostridium 5 |
| OTU136 | Bacteria | Firmicutes | Clostridia | Clostridiales | Ruminococcaceae | Ruminiclostridium 6 |
| OTU137 | Bacteria | Firmicutes | Clostridia | Clostridiales | Ruminococcaceae | Ruminiclostridium 9 |
| OTU138 | Bacteria | Firmicutes | Clostridia | Clostridiales | Ruminococcaceae | Ruminococcaceae NK4A214 group |
| OTU139 | Bacteria | Firmicutes | Clostridia | Clostridiales | Ruminococcaceae | Ruminococcaceae UCG-002 |
| OTU140 | Bacteria | Firmicutes | Clostridia | Clostridiales | Ruminococcaceae | Ruminococcaceae UCG-003 |
| OTU141 | Bacteria | Firmicutes | Clostridia | Clostridiales | Ruminococcaceae | Ruminococcaceae UCG-004 |
| OTU142 | Bacteria | Firmicutes | Clostridia | Clostridiales | Ruminococcaceae | Ruminococcaceae UCG-005 |
| OTU143 | Bacteria | Firmicutes | Clostridia | Clostridiales | Ruminococcaceae | Ruminococcaceae UCG-008 |
| OTU144 | Bacteria | Firmicutes | Clostridia | Clostridiales | Ruminococcaceae | Ruminococcaceae UCG-009 |
| OTU145 | Bacteria | Firmicutes | Clostridia | Clostridiales | Ruminococcaceae | Ruminococcaceae UCG-010 |
| OTU146 | Bacteria | Firmicutes | Clostridia | Clostridiales | Ruminococcaceae | Ruminococcaceae UCG-013 |
| OTU147 | Bacteria | Firmicutes | Clostridia | Clostridiales | Ruminococcaceae | Ruminococcaceae UCG-014 |
| OTU148 | Bacteria | Firmicutes | Clostridia | Clostridiales | Ruminococcaceae | Ruminococcus 1 |
| OTU149 | Bacteria | Firmicutes | Clostridia | Clostridiales | Ruminococcaceae | Ruminococcus 2 |
| OTU150 | Bacteria | Firmicutes | Clostridia | Clostridiales | Ruminococcaceae | Subdoligranulum |
| OTU151 | Bacteria | Firmicutes | Clostridia | Clostridiales | Ruminococcaceae | UBA1819 |
| OTU152 | Bacteria | Firmicutes | Clostridia | Clostridiales | Ruminococcaceae | [Eubacterium] coprostanoligenes group |
| OTU153 | Bacteria | Firmicutes | Clostridia | Clostridiales | Ruminococcaceae | uncultured |
| OTU154 | Bacteria | Firmicutes | Clostridia | Clostridiales | Ruminococcaceae |  |
| OTU155 | Bacteria | Firmicutes | Clostridia | Clostridiales |  |  |
| OTU156 | Bacteria | Firmicutes | Clostridia | DTU014 | uncultured bacterium | uncultured bacterium |
| OTU157 | Bacteria | Firmicutes | Erysipelotrichia | Erysipelotrichales | Erysipelotrichaceae | Candidatus Stoquefichus |
| OTU158 | Bacteria | Firmicutes | Erysipelotrichia | Erysipelotrichales | Erysipelotrichaceae | Catenibacterium |
| OTU159 | Bacteria | Firmicutes | Erysipelotrichia | Erysipelotrichales | Erysipelotrichaceae | Catenisphaera |
| OTU160 | Bacteria | Firmicutes | Erysipelotrichia | Erysipelotrichales | Erysipelotrichaceae | Dielma |
| OTU161 | Bacteria | Firmicutes | Erysipelotrichia | Erysipelotrichales | Erysipelotrichaceae | Erysipelatoclostridium |
| OTU162 | Bacteria | Firmicutes | Erysipelotrichia | Erysipelotrichales | Erysipelotrichaceae | Erysipelotrichaceae UCG-003 |
| OTU163 | Bacteria | Firmicutes | Erysipelotrichia | Erysipelotrichales | Erysipelotrichaceae | Erysipelotrichaceae UCG-006 |
| OTU164 | Bacteria | Firmicutes | Erysipelotrichia | Erysipelotrichales | Erysipelotrichaceae | Faecalitalea |
| OTU165 | Bacteria | Firmicutes | Erysipelotrichia | Erysipelotrichales | Erysipelotrichaceae | Holdemanella |
| OTU166 | Bacteria | Firmicutes | Erysipelotrichia | Erysipelotrichales | Erysipelotrichaceae | Holdemania |
| OTU167 | Bacteria | Firmicutes | Erysipelotrichia | Erysipelotrichales | Erysipelotrichaceae | Merdibacter |
| OTU168 | Bacteria | Firmicutes | Erysipelotrichia | Erysipelotrichales | Erysipelotrichaceae | Solobacterium |
| OTU169 | Bacteria | Firmicutes | Erysipelotrichia | Erysipelotrichales | Erysipelotrichaceae | Turicibacter |
| OTU170 | Bacteria | Firmicutes | Erysipelotrichia | Erysipelotrichales | Erysipelotrichaceae | [Clostridium] innocuum group |
| OTU171 | Bacteria | Firmicutes | Erysipelotrichia | Erysipelotrichales | Erysipelotrichaceae | uncultured |
| OTU172 | Bacteria | Firmicutes | Erysipelotrichia | Erysipelotrichales | Erysipelotrichaceae |  |
| OTU173 | Bacteria | Firmicutes | Negativicutes | Selenomonadales | Acidaminococcaceae | Acidaminococcus |
| OTU174 | Bacteria | Firmicutes | Negativicutes | Selenomonadales | Acidaminococcaceae | Phascolarctobacterium |
| OTU175 | Bacteria | Firmicutes | Negativicutes | Selenomonadales | Veillonellaceae | Allisonella |
| OTU176 | Bacteria | Firmicutes | Negativicutes | Selenomonadales | Veillonellaceae | Dialister |
| OTU177 | Bacteria | Firmicutes | Negativicutes | Selenomonadales | Veillonellaceae | Megamonas |
| OTU178 | Bacteria | Firmicutes | Negativicutes | Selenomonadales | Veillonellaceae | Megasphaera |
| OTU179 | Bacteria | Firmicutes | Negativicutes | Selenomonadales | Veillonellaceae | Mitsuokella |
| OTU180 | Bacteria | Firmicutes | Negativicutes | Selenomonadales | Veillonellaceae | Veillonella |
| OTU181 | Bacteria | Lentisphaerae | Lentisphaeria | Victivallales | Victivallaceae | Victivallis |
| OTU182 | Bacteria | Lentisphaerae | Lentisphaeria | Victivallales | Victivallaceae | uncultured bacterium |
| OTU183 | Bacteria | Lentisphaerae | Lentisphaeria | Victivallales | vadinBE97 | uncultured bacterium |
| OTU184 | Bacteria | Lentisphaerae | Lentisphaeria | Victivallales | vadinBE97 | uncultured rumen bacterium |
| OTU185 | Bacteria | Proteobacteria | Alphaproteobacteria | Rhodospirillales | uncultured | gut metagenome |
| OTU186 | Bacteria | Proteobacteria | Alphaproteobacteria | Rhodospirillales | uncultured | uncultured bacterium |
| OTU187 | Bacteria | Proteobacteria | Deltaproteobacteria | Desulfovibrionales | Desulfovibrionaceae | Bilophila |
| OTU188 | Bacteria | Proteobacteria | Deltaproteobacteria | Desulfovibrionales | Desulfovibrionaceae | Desulfovibrio |
| OTU189 | Bacteria | Proteobacteria | Deltaproteobacteria | Desulfovibrionales | Desulfovibrionaceae | uncultured |
| OTU190 | Bacteria | Proteobacteria | Gammaproteobacteria | Aeromonadales | Succinivibrionaceae | Succinivibrio |
| OTU191 | Bacteria | Proteobacteria | Gammaproteobacteria | Betaproteobacteriales | Burkholderiaceae | Parasutterella |
| OTU192 | Bacteria | Proteobacteria | Gammaproteobacteria | Betaproteobacteriales | Burkholderiaceae | Sutterella |
| OTU193 | Bacteria | Proteobacteria | Gammaproteobacteria | Betaproteobacteriales | Burkholderiaceae |  |
| OTU194 | Bacteria | Proteobacteria | Gammaproteobacteria | Enterobacteriales | Enterobacteriaceae | Escherichia-Shigella |
| OTU195 | Bacteria | Proteobacteria | Gammaproteobacteria | Enterobacteriales | Enterobacteriaceae | Hafnia-Obesumbacterium |
| OTU196 | Bacteria | Proteobacteria | Gammaproteobacteria | Enterobacteriales | Enterobacteriaceae | Proteus |
| OTU197 | Bacteria | Proteobacteria | Gammaproteobacteria | Enterobacteriales | Enterobacteriaceae |  |
| OTU198 | Bacteria | Proteobacteria | Gammaproteobacteria | Pasteurellales | Pasteurellaceae | Haemophilus |
| OTU199 | Bacteria | Proteobacteria | Gammaproteobacteria | Pseudomonadales | Pseudomonadaceae | Pseudomonas |
| OTU200 | Bacteria | Tenericutes | Mollicutes | Anaeroplasmatales | Anaeroplasmataceae | Anaeroplasma |
| OTU201 | Bacteria | Tenericutes | Mollicutes | Izimaplasmatales | gut metagenome | gut metagenome |
| OTU202 | Bacteria | Tenericutes | Mollicutes | Izimaplasmatales | uncultured bacterium | uncultured bacterium |
| OTU203 | Bacteria | Tenericutes | Mollicutes | Izimaplasmatales | uncultured organism | uncultured organism |
| OTU204 | Bacteria | Tenericutes | Mollicutes | Mollicutes RF39 | gut metagenome | gut metagenome |
| OTU205 | Bacteria | Tenericutes | Mollicutes | Mollicutes RF39 | uncultured Mollicutes bacterium | uncultured Mollicutes bacterium |
| OTU206 | Bacteria | Tenericutes | Mollicutes | Mollicutes RF39 | uncultured bacterium | uncultured bacterium |
| OTU207 | Bacteria | Tenericutes | Mollicutes | Mollicutes RF39 | uncultured rumen bacterium | uncultured rumen bacterium |
| OTU208 | Bacteria | Tenericutes | Mollicutes | Mollicutes RF39 |  |  |
| OTU209 | Bacteria | Verrucomicrobia | Verrucomicrobiae | Opitutales | Puniceicoccaceae | uncultured |
| OTU210 | Bacteria | Verrucomicrobia | Verrucomicrobiae | Verrucomicrobiales | Akkermansiaceae | Akkermansia |
| OTU211 | Eukaryota | SAR | Stramenopiles | Incertae Sedis | Blastocystis | Blastocystis hominis |
