# Supplementary material for: The potential of fecal microbiota and amino acids to detect and monitor patients with adenoma
Source: Gut Microbes. 2022 Feb 21;14(1):2038863. doi: 10.1080/19490976.2022.2038863 (PMC8865277; doi:10.1080/19490976.2022.2038863)
Supplement: Supplemental Material [file KGMI_A_2038863_SM4476.zip › supplementary/Supplementary Table 3 Performance matrices.docx]

Supplementary Table 3. Performance matrices of adenoma specific panel

|  | **AUC** | **Sensitivity** | **Specificity** |
| --- | --- | --- | --- |
| Training/Discovery | 0.846 (0.805- 0.888) | 0.772 (0.709 - 0.835) | 0.860 (0.808 - 0.912) |
| Test data | 0.787 (0.638 - 0.935) | 0.789 (0.789 - 0.973) | 0.737 (0.539 - 0.935) |
